# Reveal the Deformation Mechanism of (110) Silicon from Cryogenic Temperature to Elevated Temperature by Molecular Dynamics Simulation

**DOI:** 10.3390/nano9111632

**Published:** 2019-11-18

**Authors:** Jing Han, Yuanming Song, Wei Tang, Cong Wang, Liang Fang, Hua Zhu, Jiyun Zhao, Jiapeng Sun

**Affiliations:** 1 School of Mechanical and Electrical Engineering, China University of Mining and Technology, Xuzhou 221116, China; TS18050060A31@cumt.edu.cn (Y.S.); tangwei@cumt.edu.cn (W.T.); TS18050062A31@cumt.edu.cn (C.W.); zhuhua@cumt.edu.cn (H.Z.); jyzhao@cumt.edu.cn (J.Z.); 2 State Key Laboratory for Mechanical Behavior of Materials, Xi’an Jiaotong University, Xi’an 710049, China; fangl@xjtu.edu.cn; 3 College of Mechanics and Materials, Hohai University, Nanjing 210098, China

**Keywords:** single crystalline silicon, nanoindentation, molecular dynamics simulation, phase transformation, deformation mechanism, temperature

## Abstract

Silicon undergoes a brittle-to-ductile transition as its characteristic dimension reduces from macroscale to nanoscale. The thorough understanding of the plastic deformation mechanism of silicon at the nanoscale is still challenging, although it is essential for developing Si-based micro/nanoelectromechanical systems (MEMS/NEMS). Given the wide application of silicon in extreme conditions, it is, therefore, highly desirable to reveal the nanomechanical behavior of silicon from cryogenic temperature to elevated temperature. In this paper, large-scale molecular dynamics (MD) simulations were performed to reveal the spherical nanoindentation response and plastic deformation mechanism of (110)Si at the temperature range of 0.5 K to 573 K. Special attention was paid to the effect of temperature. Multiple pop-ins detected in load/pressure-indentation strain curves are impacted by temperature. Four featured structures induced by nanoindentation, including high-pressure phases, extrusion of α-Si, dislocations, and crack, are observed at all temperatures, consistent with experiment results. The detailed structure evolution of silicon was revealed at the atomic scale and its dependence on temperature was analyzed. Furthermore, structure changes were correlated with pop-ins in load/pressure-indentation strain curves. These results may advance our understanding of the mechanical properties of silicon.

## 1. Introduction

Single crystalline silicon is the most important material in the semiconductor industry and in micro/nanoelectromechanical systems (MEMS/NEMS). To facilitate the design and fabrication of silicon-based devices, it is particularly important to investigate its mechanical behavior and deformation mechanism. Recently, silicon has found wide application in extreme conditions, such as nuclear fusion technology, superconductor science and technology, and space exploration [1], which demands the deep understanding of the mechanical behavior of silicon from cryogenic temperature to elevated temperature.

In most situations, the performance and producing of MEMS/NEMS can be better reflected at the nanometer level. Hence, much effort has been devoted to revealing the nanoscale mechanical behavior and deformation mechanism of silicon. Nanoindentation is regarded as an effective method to examine nanomechanical properties. The load-depth (*P*-*h*) curve is the most straightforward result which is linked to the structure changes of the material beneath the indenter. One of the most interesting features is so-called pop-in (PI), represented by the discontinuous steps or plains in *P*-*h* curve. Previous studies indicated that one, several, or none pop-ins can be discovered in *P*-*h* curve, depending on the geometry of indenter and the peak load [2,3,4,5,6,7]. However, the definite conclusion on the occurrence of PI and its mechanism is still lack, and many inconsistent results are reported. Inspired by the results from diamond-anvil cell (DAC) experiments, the high-pressure phase transformations (HPPT) are widely accepted to be responsible for the first PI [3,4,5]. This scenario is supported by in situ electrical measurements by which a transformation from a semiconductor-like phase to a metallic phase is observed [6,7]. The resulting metallic phase is generally accepted to be the *β*-Si phase, which is a body-centered-tetragonal structure with six-fold coordination. Molecular dynamics (MD) simulations provide the direct evidence of HPPT at the atomic scale and reveal the detailed distribution, structural characteristics, and phase transformation process [8,9,10,11,12,13]. However, this scenario is continuously challenged. Bradby et al. found that the PI was linked to the simple extrusion of material beneath the indenter [14]. This result is supported by a recent MD simulation, which shows that the extrusion of the crystalline high-pressure Si-III/XII phase is contributed to the observed PI [2]. Recently, Wong et al. reported that dislocation or HPPT drives the elastic-plastic transition which are mutually exclusive of each other, occurring statistically [15]. In fact, high-pressure phases, dislocations, crack and slip bank generally coexist beneath the indenter represented by the post-mortem cross-sectional transmission electron microscopy [14,16]. Until now, it is indeed highly challenging to these deformation modes to PIs in *P*-*h* curve due to the practical obstacles in experiment.

Consequently, the mechanism behind the easily perceived PIs in *P*-*h* curve is still unavailable. The full understanding of the deformation mechanism is still a challenging issue and thus an interesting research topic. Most previous studies were conducted on the (100)Si and at room temperature. Although crystallographic orientation and temperature have been shown to affect significantly the nanomechanical behavior of Si, a religious understanding of their influence is still unavailable. In this paper, large-scale MD simulations were performed to reveal the spherical nanoindentation response of (110)Si at a temperature range of 0.5 K to 573 K. The nanomechanical behavior and deformation mechanism were investigated at atomic scale. Special attention was paid to the effect of temperature. The results showed multiple pop-ins in the *P*-*h* curve depending on peak load and temperature. Three types of HPPTs and extrusion of α-Si were identified to be contributed to pop-ins. Furthermore, the correlation between deformation modes and pop-ins was established. The effects of crystallographic orientation and temperature on deformation mechanism of silicon were analyzed based on present MD simulation results and available data in references, which advances our understanding of the mechanical properties of silicon.

## 2. Materials and Methods

**Figure 1:**
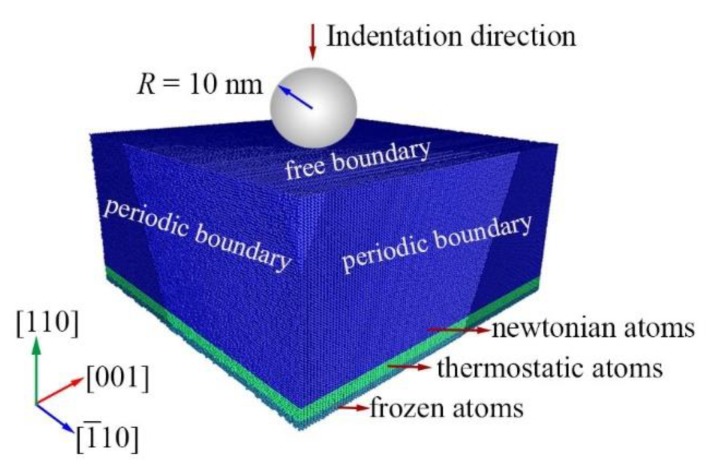
Molecular dynamics model.

Nanoindentations on the (110) Si were simulated by large-scale MD simulation at a temperature range of 0.5 K to 573 K. A new screened empirical bond-order potential (a screened version of potential developed by Erhart et al. [17] and Moseler et al. [18]) was used to model the interaction between Si atoms, which is suitable to describe the deformation mechanisms during nanoindentation, cutting of monocrystalline, and polycrystalline silicon under uniaxial tension [11,19,20]. The virtual spherical indenter with a diameter of 20 nm was used, which is modeled using a repulsive force: $F=AH\left(r\right){\left(R-r\right)}^{2}$ where *A* is force constant with a value of 10 eV/A^2^, *H*(*r*) is a step function, *R* is the indenter radius, and *r* is the distance from the silicon atoms to the indenter sphere center. A typical MD mode of nanoindentation was employed (Figure 1) which can be commonly found in references [11,21]. This mode was composed of the frozen layer with a thickness of 0.5 nm which provided structural stability, the thermostatic layer with a thickness of 2 nm which dissipated excessive thermal energy, and the Newtonian layer. The initial cubic (110)Si sample has a dimension of 65.17 × 65.21 × 31.99 nm^3^, which contains more than 6.5 million Si atoms. The silicon atoms were initially arranged in a diamond cubic structure with a lattice parameter of 0.5431 nm. Then, the sample underwent a meticulous heat treatment to get the equilibrium configuration at a specified temperature, which was described in detail in our previous paper [19].

Serials of MD simulations of nanoindentation were performed at a constant velocity of 0.08 nm/ps by the LAMMPS code [22]. Free boundary condition was applied along the indentation direction, while periodic boundary condition was applied along the other two directions. During nanoindentation, the thermostatic layer was maintained a constant specified temperature to dissipate excess thermal energy, using the NVT ensemble with Langevin Dynamics [23]. All the atoms in the Newtonian layer were free to move according to the Newtonian motion equations. The input file for the LAMMPS code to simulate the nanoindentation on the (110)Si at a temperature of 300 K can be found in Supplementary Materials.

After indentation, the high-pressure phases and dislocations were identified and extracted from the atom trajectories. The combination of the modified coordination number (MCN) considering the first and second nearest neighbors, the radial distribution function (RDF), and the bond-angle distribution function (ADF) were applied to identify the phases (Si-I, Si-II, Si-III, Si-XIII, bct5, and α-Si). The detailed description of this combination method can be found in our previous works [8,19]. The dislocation was identified using the slip vector analysis (SV) and the dislocation extraction algorithm (DXA) [24].

## 3. Results and Discussion

### 3.1. Mechanical Behavior

**Figure 2:**
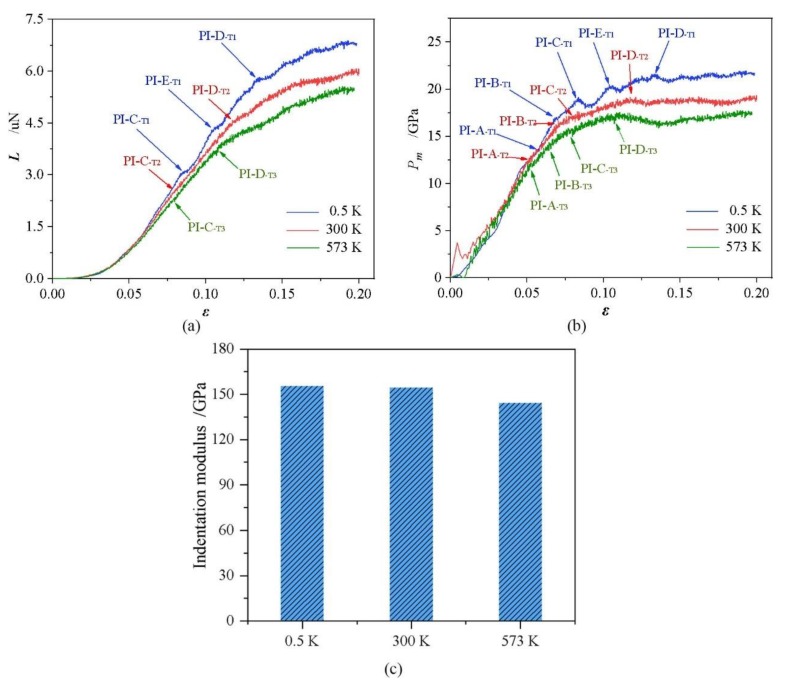
(**a**) *L*-*ε* curves, (**b**) *P_m_*-*ε* curve, and (**c**) indentation modulus under nanoindentation of Si(110) at different temperatures, where *L* is load, *ε* is indentation strain, *P_m_* is mean contact pressure, PI is pop-in.

Figure 2a,b show the load-indentation strain (*L*-*ε*) and pressure-indentation strain (*P_m_*-*ε*) curves of (110) Si under nanoindentation at three different temperatures. For better comparison and evaluation, the characteristic indentation strain *ε* = 0.2*h*/*a* was adopted to replace the generally used indentation depth *h*, where *a* is the contact radius. The mean contact pressure is expressed as *P_m_* = *L*/π*a*^2^, where *L* is the applied load. This figure highlights a temperature-dependent mechanical behavior of (110)Si under nanoindentation. During the elastic loading, the *L*-*h* curves well overlap with Hertzian law [25]:

(1) $$L=\frac{4}{3}Mh^{\frac{3}{2}}\sqrt{R}$$

where *L* is the indentation load, *h* is the indentation depth, a is the *R* is the radius of the indenter, and the so-called indentation modulus *M* describes the elastic response of materials, which can be obtained by fitting the *L*-*h* curves in elastic deformation. The fitted indentation modulus, representing the material’s elastic response under nanoindentation, decreases with the increase of temperature, as shown in Figure 2c. The fluctuation of the *L*-*ε* and *P_m_*-*ε* curves become larger at an elevated temperature due to intensive atomic thermal vibration. Moreover, the load and contact pressure decrease monotonously with the increase in temperature as expected, which is more remarkable at a large *ε*. This implies a decreased hardness with the increase in temperature. Overall, *P_m_* continuously increases and tends to be a stable value as a large *ε* is reached at all temperatures.

**Figure 3:**
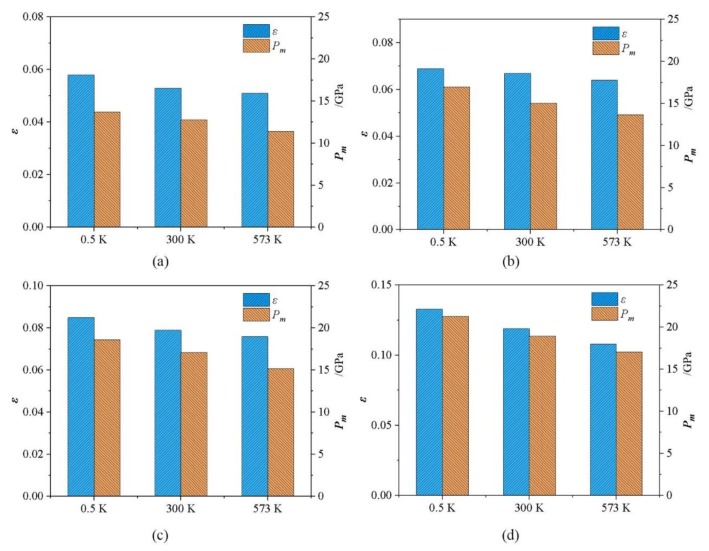
The indentation strain and contact pressure for (**a**) PI-A, (**b**) PI-B, (**c**) PI-C, and (**d**) PI-D depending on temperature.

One key result is that multiple PIs are observed in the *L*-*ε* and *P_m_*-*ε* curves, and are significantly influenced by temperature, as shown in Figure 2a,b. Four PIs are detected in the *P_m_*-*ε* curves when nanoindentation is conducted at 300 K and 573 K, whereas only two corresponding PIs are visible in the *L*-*ε* curves. These PIs hereafter are referred to as PI-A, PI-B, PI-C, and PI-D (the corresponding indentation strain increases from PI-A to PI-D). As the temperature decreases to 0.5 K, an additional PI-E appears in the *L*-*ε* and *P_m_*-*ε* curves between the PI-C and PI-D besides the other four PIs. The PI-A is the first PI and thus indicates the onset of plastic deformation. The subsequent PIs imply the structure changes of the material under indenter. Figure 3 shows the indentation stain and contact pressure for PIs (PI-A, PI-B, PI-C, and PI-D) as a function of temperature. It can be observed that the indentation strain and contact pressure for every PI decrease with an increase in temperature, which indicates that the occurrence of PIs is earlier when temperature rises. The elevated temperature lowers the energy barrier for the structure change and thus results in low load or contact pressure for PIs.

### 3.2. The Structure Changes Induced by Nanoindentation

**Figure 4:**
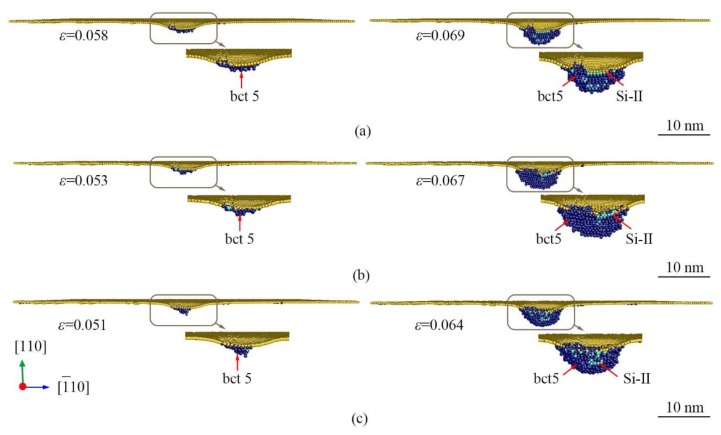
The longitudinal section view of phase distribution induced by nanoindentation on (110)Si near the PI-A and PI-B (as shown in Figure 2c) at the temperature of (**a**) 0.5 K, (**b**) 300 K, and (**c**) 573 K. The atoms are color-coded by MCN. The dark blue, lawn green, and yellow atoms are Si-II, bct5, and surface atoms, respectively.

Figure 4 shows the high-pressure phase evolution of (110)Si under nanoindentation near the PI-A. The HPPT from Si-I to bct5 initiates at the indentation strain of 0.058, 0.053, and 0.051 at 0.5 K, 300 K, and 573 K, respectively. This transformation means the onset of plastic deformation. The high-pressure phase Si-II is first visible at the indentation strain of 0.069, 0.067, and 0.064, at 0.5 K, 300 K, and 573 K, respectively. This result indicates that the HPPT of Si-I to bct5 occurs ahead of that of Si-I to Si-II. As can be seen from the *P_m_*-*ε* curves in Figure 2b, the HPPTs from Si-I to bct5 and from Si-I to Si-II induce small PI-A and PI-B at all temperatures, respectively. PI-A and PI-B are inconspicuous in *P_m_*-*ε* and are not obvious in the *L*-*ε* curves, especially at elevated temperatures. Our previous studies suggested that the HPPT from Si-I to Si-II contributes to the first PI on (100)Si [19], whereas the HPPT from Si-I to *α*-Si leads to the first PI on (111)Si [10]. In comparison to the markable PI in both the *L*-*ε* and *P_m_*-*ε* curves for (100)Si and (111)Si, the two inconspicuous PIs induced by HPPT from Si-I to bct5 and from Si-I to Si-II are only visible in the *P_m_*-*ε* curve in the case of (110)Si. This implies that the volume of the resultant high-pressure phases (Si-II and bct5) is so small that load relaxation is slow, resulting in the inconspicuous PIs.

**Figure 5:**
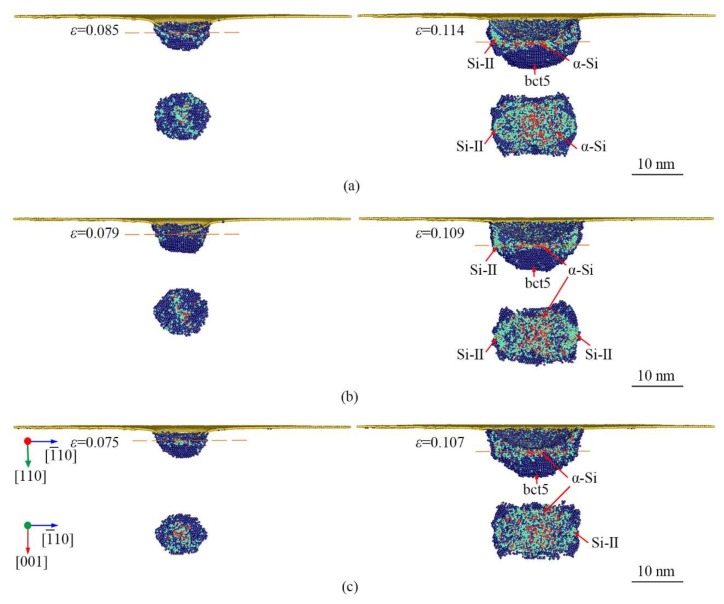
The longitudinal section and cross-section view of phase distribution induced by nanoindentation on (110)Si near the PI-C at the temperature of (**a**) 0.5 K, (**b**) 300 K, and (**c**) 573 K. The atoms are color-coded by MCN. The dark blue, lawn green, and yellow atoms are Si-II, bct5, and surface atoms, respectively.

With the increasing indentation strain or applied load, *α*-Si begins to be visible underneath the indenter at *ε* = 0.085, 0.079, and 0.076 at 0.5 K, 300 K, and 573 K, respectively, as shown in Figure 5. The *α*-Si region surrounded by Si-II and bct5 regions expand continuously as the increase in indentation strain as well as Si-II and bct5 regions. Thus, the *α*-Si is transformed from Si-II and bct5. This HPPT triggers the first visible PI, i.e., PI-C in the *L*-*ε* curves at all temperatures. Interestingly, the *α*-Si region is much larger than the Si-II and bct5 regions at all temperatures.

**Figure 6:**
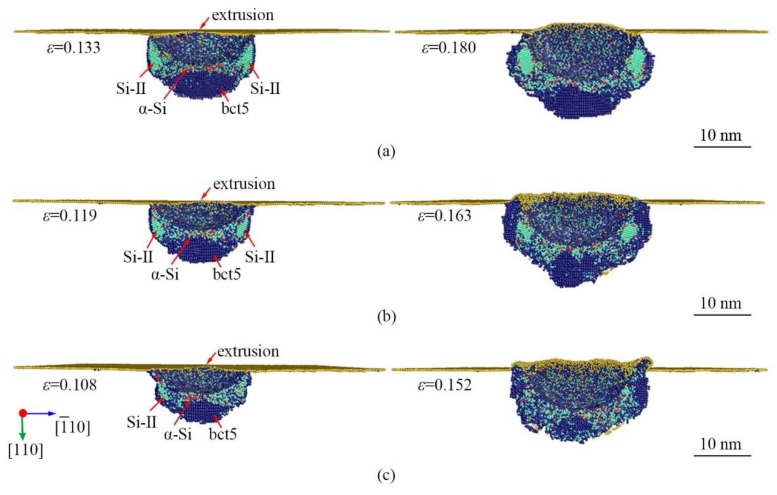
The longitudinal section view of phase distribution induced by nanoindentation on (110)Si near the PI-D at the temperature of (**a**) 0.5 K, (**b**) 300 K, and (**c**) 573 K. The atoms are color-coded by MCN. The dark blue, lawn green, and yellow atoms are Si-II, bct5, and surface atoms, respectively.

With the increase in indentation strain, the Si-II, bct5, and *α*-Si expand continuously, and no other new phase is generated at all temperatures. When the indentation strain reaches to *ε* = 0.133, 0.119, and 0.108 at 0.5 K, 300 K, and 573 K, respectively, the sudden extrusion of α-Si outside the indentation cavity is observed, as indicated in Figure 6. The extrusion forms the pileup composed of α-Si in the surface, which indicates that the transformation region extends beyond the constraint of the indenter. The volume of pileup enlarges with the increase in nanoindentation strain. The increase of indentation strain causes the structure distortion and the reduction in volume that is ascribed to the reduction of the atom spacing [26]. When the volume reduction caused by structural deformation is insufficient to accommodate the volume pushed by the indentation, the newly generated α-Si will be extruded from the indentation cavity. The sudden extrusion induces a rapid change in internal stress and thus generates the PI-D in both *L*-*ε* and *P_m_*-*ε* curves. From the *P_m_*-*ε* curve, the mean contact pressure tends to be a contrast value after the PI-D.

**Figure 7:**
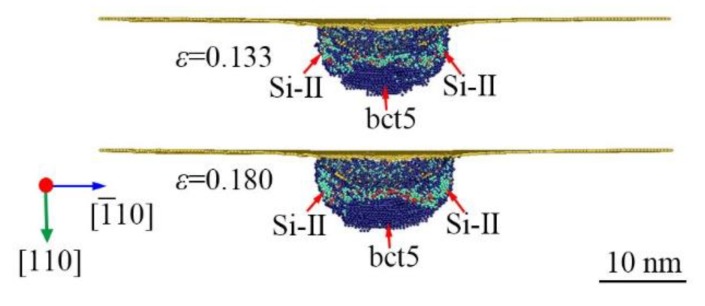
The longitudinal section view of phase distribution induced by nanoindentation on (110)Si near the PI-E at 573 K. The atoms are color-coded by MCN. The dark blue, lawn green, and yellow atoms are Si-II, bct5, and surface atoms, respectively.

There is an additional PI in both the *L*-ε and *P_m_*-*ε* curve at 0.5 K between the PI-C and PI-D, as indicated in Figure 2 and Figure 7 shows the phase morphology evolution near the PI-E. The PI-E can be linked to the sudden increase in Si-II volume. Because of the low kinetic energy of atoms at 0.5 K, the slight structure changes could lead to the undulation of the *L*-ε and *P_m_*-*ε* curves. Indeed, the expansion of Si-II is accelerated between the PI-C and PI-D as shown in Figure 4 and Figure 5, whereas no visible PI can be generated in the *L*-ε and *P_m_*-*ε* curves.

### 3.3. Dislocations and Crack

**Figure 8:**
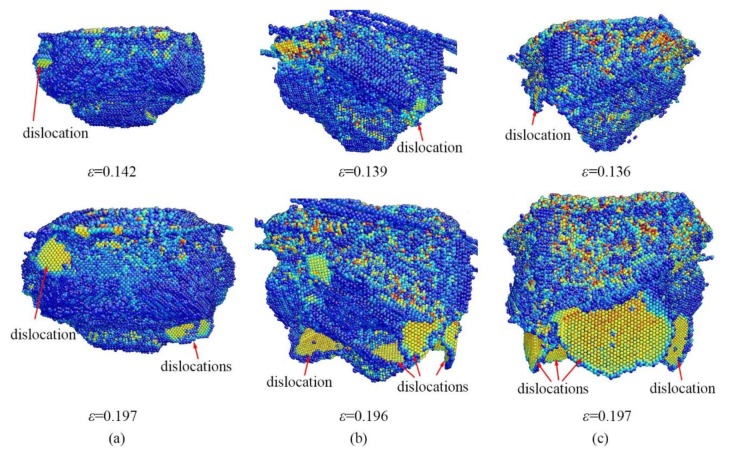
The development of dislocations at (**a**) 0.5 K, (**b**) 300 K, and (**c**) 573 K. The atoms are colored by SV.

Besides HPPTs, the dislocations are also found responsible for the plastic deformation. Figure 8 displays the evolution of dislocations during nanoindentation at three given temperatures. The dislocation loops are found to nucleate on the high-pressure phases/Si interface at the indentation strain of 0.142, 0.139, and 0.136 at 0.5 K, 300 K, and 573 K. All the dislocations are identified as perfect ½<110>{111} dislocations according to both the SV analysis and DXA. However, the dislocation nucleation and slipping does not induce a PI in the *L*-*ε* and *P_m_*-*ε* curves. The indentation strain for dislocation nucleation decreases with the increase in temperature, indicating early activation of dislocation at an elevated temperature. In addition, the extrusion of α-Si happens before the occurrence of dislocations at all temperatures. As the indentation processes, the dislocations slip and multiply continuously. As a result, many dislocations can be found underneath the transformation region, and the higher the temperature is, the more the dislocations are. However, the contribution of dislocations to plastic deformation is relatively weak because of insufficient dislocations compared to the metals in which dislocation dominates the plastic deformation [25,27,28,29,30,31].

**Figure 9:**
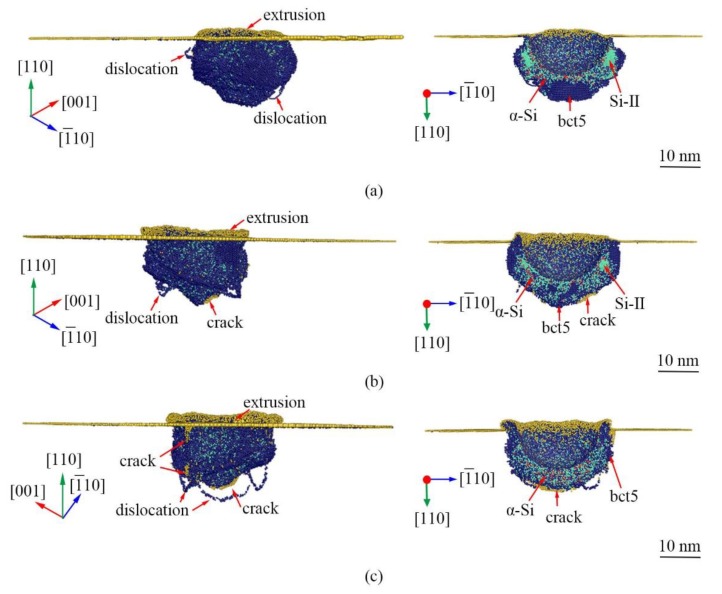
The microstructure morphology of (110)Si at (**a**) 0.5 K at *ε* = 0.197, (**b**) 300 K at *ε* = 0.196, and (**c**) 573 K at *ε* = 0.197. The atoms are color-coded by MCN. The dark blue, lawn green, and yellow atoms are Si-II, bct5, and surface atoms, respectively.

The microstructures of (100)Si induced by nanoindentation at a large indentation strain depending on temperature are displayed in Figure 9. Our MD simulations predict several featured structures including HPPT region surrounding indenter, extrusion of α-Si in the surface, dislocations, and crack in the high-pressure phases/Si interface, which involves almost all the observed structure features of Si induced by nanoindentation and is coincided with the experiment results [14,32,33]. Bct5, Si-II, and α-Si are high-pressure phases. α-Si surrounds indenter, and the bct5 is on the edge of the transformation regions. The volume of Si-II is very small, especially at an elevated temperature. The volume of *α*-Si is relatively large and increases with the increase in temperature. In comparison to (100)Si, (110)Si and (111)Si have large *α*-Si region but small Si-II region. The detailed structure features depend strongly on the crystallographic orientation. Consequently, MD simulations render us a complete scenario of structure changes in Si induced by nanoindentation using a spherical indenter.

In addition, some surface atoms are found directly adjacent to the transformation region at the large indentation strain and at a temperature of 300 K and 573 K, as shown in Figure 9b,c, indicting the cracks. The cracks nucleate on the high-pressure phases/Si interface, where a large stress concentration is present due to the discontinuous structure. The cracks are generally found in experiments when nanoindentation is applied to Si at a large load or indentation strain [16]. Therefore, Si is generally regarded as a brittle material.

## 4. Conclusions

Aiming to reveal nanomechanical behavior of silicon from cryogenic temperature to elevated temperature, a series of large-scale MD simulations were performed to investigate the spherical nanoindentation response of (110)Si at a temperature range of 0.5 K to 573 K. The main conclusions were drawn as follows:

(1) Multiple PIs which depend on temperature are observed in the *L*-*ε* and *P_m_-ε* curves. Cryogenic temperature facilitates the occurrence of PIs and thus results in more PIs compared to elevated temperature. More PIs can be found in the *P_m_*-*ε* curves than in the *L*-*ε* curves.
(2) The four featured structure including high-pressure phases surrounding indenter, extrusion of *α*-Si in the surface, dislocations, crack in the high-pressure phases/Si interface are found. Even so, the HPPT is still the dominated plastic deformation mechanism during nanoindentation with a small indenter in the present condition.
(3) The high-pressure phases of bct5, Si-II, and *α*-Si occur beneath the indenter in turn and dominate the plastic deformation. The volume of Si-II is very small, especially at an elevated temperature. In contrast, the volume of *α*-Si is relatively large and increases with the increase in temperature.
(4) The perfect ½<110>{111} dislocation loops are found to nucleate on the high-pressure phases/Si interface at large indentation strains at the present condition. The higher the temperature is, the more the dislocations are. However, the contribution of dislocations to plastic deformation is relatively weak because of insufficient dislocations, so that the dislocation nucleation and slipping do not induce a PI in the *L*-ε and *P_m_*-*ε* curves.
(5) The formations of bct5, Si-II, and *α*-Si are responsible for the PIs at small indentation strain. The extrusion of *α*-Si contributes to the PI at high indentation strains. However, the dislocation and crack processing do not lead to a PI.

The present result shows a complete scenario of the PIs, the structure changes, and mechanical behavior of Si induced by nanoindentation from cryogenic temperature to elevated temperature, which advances our understanding of the mechanical properties of silicon materials.

## Supplementary Materials

The following are available online at https://www.mdpi.com/2079-4991/9/11/1632/s1.

## References

[1] Judy J.W. (2001). Microelectromechanical systems (MEMS): Fabrication, design and applications. Smart Mater. Struct..

[2] Abram R., Chrobak D., Nowak R. (2017). Origin of a Nanoindentation Pop-in Event in Silicon Crystal. Phys. Rev. Lett..

[3] Juliano T., Domnich V., Gogotsi Y. (2004). Examining pressure-induced phase transformations in silicon by spherical indentation and Raman spectroscopy: A statistical study. J. Mater. Res..

[4] Zhang Z., Stukowski A., Urbassek H.M. (2016). Interplay of dislocation-based plasticity and phase transformation during Si nanoindentation. Comput. Mater. Sci..

[5] Li C., Zhang L. (2009). Mechanical behavior characterisation of silicon and effect of loading rate on pop-in: A nanoindentation study under ultra-low loads. Mater. Sci. Eng. A.

[6] Clarke D.R., Kroll M.C., Kirchner P.D., Cook R.F., Hockey B.J. (1988). Amorphization and conductivity of silicon and germanium induced by indentation. Phys. Rev. Lett..

[7] Gerbig Y.B., Michaels C.A., Forster A.M., Cook R.F. (2012). In situ observation of the indentation-induced phase transformation of silicon thin films. Phys. Rev. B.

[8] Sun J., Fang L., Han J., Han Y., Chen H., Sun K. (2014). Phase transformations of mono-crystal silicon induced by two-body and three-body abrasion in nanoscale. Comput. Mater. Sci..

[9] Sun J., Ma A., Jiang J., Han J., Han Y. (2016). Orientation-dependent mechanical behavior and phase transformation of mono-crystalline silicon. J. Appl. Phys..

[10] Han J., Xu S., Sun J., Fang L., Zhu H. (2017). Pressure-induced amorphization in the nanoindentation of single crystalline silicon. RSC Adv..

[11] Han J., Sun J., Xu S., Song D., Liu H., Han Y., Fang L. (2018). Deformation mechanisms at multiple pop-ins under spherical nanoindentation of (1 1 1) Si. Comput. Mater. Sci..

[12] Kim D.E., Oh S.I. (2008). Deformation pathway to high-pressure phases of silicon during nanoindentation. J. Appl. Phys..

[13] Kim D.E., Oh S.I. (2006). Atomistic simulation of structural phase transformations in monocrystalline silicon induced by nanoindentation. Nanotechnology.

[14] Bradby J.E., Williams J.S., Wong-Leung J., Swain M.V., Munroe P. (2000). Transmission electron microscopy observation of deformation microstructure under spherical indentation in silicon. Appl. Phys. Lett..

[15] Wong S., Haberl B., Williams J.S., Bradby J.E. (2015). The influence of hold time on the onset of plastic deformation in silicon. J. Appl. Phys..

[16] Zarudi I., Zhang L.C., Cheong W., Yu T.X. (2005). The difference of phase distributions in silicon after indentation with Berkovich and spherical indenters. Acta Mater..

[17] Albe K., Erhart P. (2005). Analytical potential for atomistic simulations of silicon, carbon, and silicon carbide. Phys. Rev. B.

[18] Pastewka L., Klemenz A., Gumbsch P., Moseler M. (2013). Screened empirical bond-order potentials for Si-C. Phys. Rev. B.

[19] Sun J., Li C., Jing H., Ma A., Fang L. (2017). Nanoindentation induced deformation and pop-in events in a silicon crystal: Molecular dynamics simulation and experiment. Sci. Rep..

[20] Goel S., Kovalchenko A., Stukowski A., Cross G. (2016). Influence of microstructure on the cutting behaviour of silicon. Acta Mater..

[21] Lin Y.H., Chen T.C., Yang P.F., Jian S.R., Lai Y.S. (2017). Atomic-level simulations of nanoindentation-induced phase transformation in mono-crystalline silicon. Appl. Surf. Sci..

[22] Plimpton S.J. (1995). Fast Parallel Algorithms for Short-range Molecular-Dynamics. J. Comput. Phys..

[23] Schneider T., Stoll E. (1978). Molecular-dynamics study of a three-dimensional one-component model for distortive phase transitions. Phys. Rev. B.

[24] Stukowski A., Bulatov V.V., Arsenlis A. (2012). Automated identification and indexing of dislocations in crystal interfaces. Model. Simul. Mater. Sci. Eng..

[25] Sun J., Fang L., Sun K., Han J. (2011). Direct observation of dislocations originating from perfect twin boundaries. Scr. Mater..

[26] Jian S. (2008). Mechanical deformation induced in Si and GaN under Berkovich nanoindentation. Nanoscale Res. Lett..

[27] Sun J., Yang Z., Liu H., Han J., Wu Y., Zhuo X., Song D., Jiang J., Ma A., Wu G. (2019). Tension-compression asymmetry of the AZ91 magnesium alloy with multi-heterogenous microstructure. Mater. Sci. Eng. A.

[28] Sun J., Fang L., Ma A., Jiang J., Han Y., Chen H., Han J. (2015). The fracture behavior of twinned Cu nanowires: A molecular dynamics simulation. Mater. Sci. Eng. A.

[29] Han J., Fang L., Sun J., Han Y., Sun K. (2012). Length-dependent mechanical properties of gold nanowires. J. Appl. Phys..

[30] Sun J., Yang Z., Han J., Liu H., Song D., Jiang J., Ma A. (2018). High strength and ductility AZ91 magnesium alloy with multi-heterogenous microstructures prepared by high-temperature ECAP and short-time aging. Mater. Sci. Eng. A.

[31] Sun J., Xu B., Yang Z., Zhou H., Han J., Wu Y., Song D., Yuan Y., Zhuo X., Liu H. (2019). Achieving excellent ductility in high-strength Mg-10.6Gd-2 Ag alloy via equal channel angular pressing. J. Alloys Compd..

[32] Ruffell S., Bradby J.E., Williams J.S., Munroe P. (2007). Formation and growth of nanoindentation-induced high pressure phases in crystalline and amorphous silicon. J. Appl. Phys..

[33] Saka H., Shimatani A., Suganuma M., Suprijadi (2002). Transmission electron microscopy of amorphization and phase transformation beneath indents in Si. Philos. Mag. A.

