# Supplementary material for: Reveal the Deformation Mechanism of (110) Silicon from Cryogenic Temperature to Elevated Temperature by Molecular Dynamics Simulation
Source: Nanomaterials (Basel). 2019 Nov 18;9(11):1632. doi: 10.3390/nano9111632 (PMC6915669; doi:10.3390/nano9111632)
Supplement: Supplementary file 1 [file nanomaterials-09-01632-s001.pdf]

The input file for the LAMMPS code to simulate the nanoindentation on the (110)Si at a temperature of 300 K is listed as follows:

```
units            metal
boundary         p m p
atom_style       atomic
neighbor         4 bin
neigh_modify     delay 5
lattice          diamond 5.431
read_data        data.txt
pair_style       atomistica BrennerScr
pair_coeff       * *    Si Si Si
group subin      type 1
group fixatom    type 2
group thermatom  type 3
group            sub union subin fixatom thermatom
fix              subin nve
fix              6 fixatom setforce 0 0 0
fix              7 fixatom nve
velocity         fixatom set 0 0 0 units box
fix              9 thermatom langevin 300 300 0.1 89994827 tally yes
fix              10 thermatom nve
variable         yy equal "408.70-step*0.002"
fix              8 all indent 10.0 sphere 325.86 v_yy 326.041 100 units box
thermo           50
timestep         0.0025
thermo_style     custom elapsed temp f_8[1] f_8[2] f_8[3]
dump             1 all cfg 500 dump.indentation.*.cfg mass type xs ys zs type vx vy vz
dump_modify      1 element Si Si Si
dump_modify      1 first yes
dump_modify      1 sort id
run              30000
```
